# A Systematic Approach to Providing COVID-19 Vaccinations in the Community by Student Pharmacists

**DOI:** 10.3390/pharmacy10040093

**Published:** 2022-07-30

**Authors:** Alex J. Luli, Candis M. Morello, Sarah M. Lorentz, Mark Bounthavong, Katharina Brandl, Laura A. Hart

**Affiliations:** Skaggs School of Pharmacy and Pharmaceutical Sciences, University of California San Diego, 9500 Gilman Drive, La Jolla, CA 92093, USA; cmmorello@health.ucsd.edu (C.M.M.); slorentz@health.ucsd.edu (S.M.L.); mbounthavong@health.ucsd.edu (M.B.); kbrandl@health.ucsd.edu (K.B.); l1hart@health.ucsd.edu (L.A.H.)

**Keywords:** COVID-19, immunization, community, pharmacy, students

## Abstract

Doctor of Pharmacy (PharmD) students and faculty at University of California, San Diego Skaggs School of Pharmacy and Pharmaceutical Sciences (SSPPS) were highly motivated to support local and regional COVID-19 vaccination efforts, which began in January 2021. A system was created to streamline requests for SSPPS volunteers, maximize opportunities for student learning and engagement, and ensure adherence to pharmacy practice standards and laws in the process of assisting with vaccination efforts in the community. An existing model for approving student organized events was modified to fit additional needs for COVID-19 vaccination efforts by SSPPS students and faculty. For each event, students completed a standardized form containing event details including location, date, time, pharmacist preceptors, and duties. All requests were screened by designated SSPPS faculty to ensure student safety, availability, and feasibility. After each event, students and faculty completed a unique online form designed to track volunteer hours. Students received course credit for volunteering and completing a standardized self-reflection. Comments from students’ reflections (*n* = 74) were analyzed to identify common challenges. Between 11 January 2021 and 31 May 2021, SSPPS faculty and students volunteered for 245 shifts, totaling 1346 h. Students encountered several logistical challenges, such as availability of vaccines. The system utilized allowed for SSPPS students and faculty to play an integral role in COVID-19 vaccination efforts throughout the region.

## 1. Introduction

Responding to the coronavirus disease 2019 (COVID-19) pandemic, in December 2020, the United States (U.S.) Food and Drug Administration (FDA) issued emergency use authorization for the Pfizer-BioNTech COVID-19 vaccine in individuals aged 16 years and above [1]. Shortly after, vaccines from Moderna and Janssen were also authorized for emergency use in December 2020 and February 2021, respectively [2,3]. With over 330,000 probable deaths due to COVID-19 by the end of 2020 in the U.S., there was an urgent need to quickly distribute the vaccine to eligible individuals [4]. Subsequently, there was high demand for trained volunteers to assist with vaccination efforts nationwide.

University of California, San Diego Skaggs School of Pharmacy and Pharmaceutical Sciences (SSPPS) is the only pharmacy school in California’s second most populous county. SSPPS Doctor of Pharmacy (PharmD) students and faculty were highly motivated to support local and regional COVID-19 vaccination efforts, which began in January 2021. Due to our school’s small student body (approximately 260 students) coupled with logistical challenges in procuring authorized vaccines, we decided to support numerous partner organizations with COVID-19 immunization efforts, as opposed to operating our own immunization clinics. Various organizations, ranging from community pharmacies to large health systems, were enthusiastic to receive support from SSPPS. 

A systematic process (“the System”) was created to streamline external requests for SSPPS volunteers, maximize opportunities for student learning and engagement, and ensure compliance to pharmacy practice standards and laws, in the process of assisting with vaccination efforts. Further, the System tracked vaccine activities across all SSPPS volunteers and helped facilitate a variety of volunteer opportunities in diverse settings. Here, we describe the System employed at SSPPS, including benefits, challenges, and key factors for success. In doing so, we aim to help similar academic institutions that wish to utilize pharmacy students to support critical public health measures, such as vaccination programs, while also providing quality learning experiences and avenues for community engagement.

## 2. Materials and Methods

**Figure 1:**
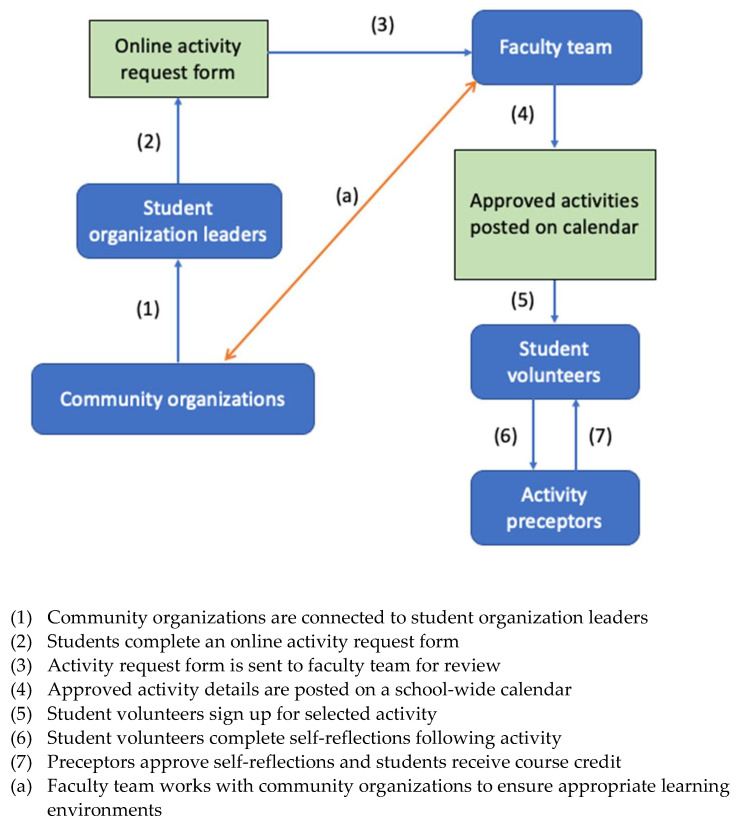
Overall process for approval of Co-curricular and IPPE activities.

The System was built on an existing framework designed to approve student activities within the SSPPS curriculum. Following guidance from the Accreditation Council for Pharmacy Education (ACPE) Accreditation Standards, the SSPPS PharmD curriculum requires that student pharmacists complete Introductory Pharmacy Practice Experiences (IPPEs) during the Pre-Advanced Pharmacy Practice Experience (Pre-APPE) curriculum. This includes at least 50 h of patient care related community service and/or outreach activities (e.g., vaccination clinics). In addition, students are required to participate in activities within the Co-Curricular (CC) course, a one-unit mandatory course each year that aims to supplement the curriculum with a focus on selected educational domains. Most activities are planned by student organizations or individual students and overseen by a faculty team responsible for ensuring all activities meet intended learning objectives under the educational domain(s). For any new CC or IPPE activity, students complete a mandatory, online request form with information about the activity including location, date, proposed learning objectives, name(s) of pharmacist preceptor(s), type of training requirements, and additional legal and clinical requirements if the activity involves direct patient care. The online request form was created using a third-party survey tool and maintained by the faculty team. All students have access to the request form via a hyperlink that is posted within course materials for both IPPE and CC courses. The faculty team provides oversight for how students can safely and efficiently sponsor their activity and is responsible for final activity approval. Approved activities are shared on a school-wide calendar, where students may sign up to participate. Students receive IPPE and/or CC course credit by participating in an activity and completing a self-reflection that requires review and approval by the activity preceptor(s). A visual schematic of this process is shown in Figure 1.

We adapted this *existing* framework for IPPE and CC activities, into the System that would encompass COVID-19 vaccine efforts. The same online activity request form was utilized, with additional information requested specifically for COVID-19 vaccination activities. This included information on current public health guidance for masking, social distancing, disinfecting, contact tracing, and viral testing. The Director of IPPE assumed the primary responsibility of reviewing this information with students, preceptors, and community partners. Additional modifications included weekly email updates directly to students that contained updated information about COVID-19 vaccines and pharmacy regulations. For example, the California State Board of Pharmacy modified existing law to allow a licensed pharmacist to oversee additional pharmacy interns when participating solely in immunization efforts [5]. This allowed more students to sign up for activities without an additional pharmacist preceptor, further expanding our capacity to provide immunization services. In addition to weekly emails, the faculty team also added this new information into the online request form for students. The System also included oversight by faculty to ensure events were safe and complied with pharmacy laws and regulations; this also extended to external organizations. Faculty were able to clarify areas of concern, request modifications, and ultimately decline an event that was not following acceptable practice standards. Additionally, the Director of IPPE was “on call” at any time for students that had urgent needs at their vaccination activity. Finally, the System maintained the existing structure that places responsibility on student organizers for submitting all required information for activity approval. This helped reduce the amount of faculty and staff effort to find new vaccine opportunities for students. 

To assess the quality of student experiences during COVID-19 vaccine activities, we reviewed their self-reflection responses to the following prompt: “List one challenge related to this activity, and strategies to overcome this challenge. Describe how you designed and implemented (or would do so in the future) solutions to this challenge.” Two independent reviewers (MB and KB) examined the responses from pharmacy students and identified statements that aligned with the World Health Organization’s Behavioural and Social Drivers (BeSD) Framework of vaccine uptake, specifically the domain of Practical Issues, which includes the subdomains of Availability, Ease of Access, and Service Quality [6]. The two reviewers coded student reflections to the Practical Issues domain of the BeSD Framework and selected statements that represented one of the subdomains. Any disagreements were resolved through discussion and re-review of the student reflections. This preliminary qualitative analysis is part of a larger study on thematic content analysis, which is currently underway. Partial results of this analysis are presented here. 

This study was exempted from full review by the institutional review board of the University of California, San Diego.

## 3. Results

### 3.1. Vaccination Efforts

SSPPS successfully partnered with 8 distinct organizations, including University of California affiliated health systems, large chain pharmacies, independent pharmacies, and non-profit community organizations. Overall, between 11 January 2021 and 31 May 2021, SSPPS faculty and students completed 245 volunteer shifts totaling 1346 h. This included 96 SSPPS students contributing a total of 1222 h (Table 1). Students from the graduating class of 2024 (2024 cohort) provided approximately 68% of the total vaccine effort; the 2023, 2022, and 2021 cohorts provided 26%, 5%, and 2%, respectively, of the total community vaccine effort from SSPPS students. Additionally, seven faculty members contributed approximately 124 h during the same period.

### 3.2. Student Self-Reflections

We collected students’ perceptions of their experiences by asking questions about the challenges they faced and how they overcame these.

Students identified non optimal workflow issues which resulted in vaccine administration delays. For example, one student highlighted that preparing doses of the COVID-19 vaccine was not performed at the same rate as administration, which led to a delay in vaccine administration. This also resulted in other providers having to assist with preparing doses,

“*We had to wait for syringes to be prefilled, and vaccinating faster than the loading the syringes. Some health care providers closed down their station to help with filling syringes before reopening their station to vaccinate*”.

—Pharmacy Student B (class of 2024)

During their time as a volunteer, SSPPS students provided consultations and education to patients receiving the COVID-19 vaccine. However, there was a shortage of printed educational information to assist with this task. One student identified a lack of COVID-19-related printed materials as a challenge during their vaccination experience,

“*I almost had trouble keeping up with the vaccinators because I spent a considerable amount of time educating patients and helping them sign up […] I wish we could have provided patients with pamphlets on the side effects, V-safe, and instructions on signing up for their second dose, as it was a bit confusing to navigate the website. If we provided patients with those documents, I would have had more time to address patient concerns, check-in with them during the 15 min wait, and chat with them. Many patients had issues signing up, so I had to figure it out and walk them through it*”.

—Pharmacy Student C (class of 2024)

One student commented that in the absence of available COVID-19 vaccines, they were still able to administer another appropriate vaccine. They also supported COVID-19 testing efforts,

“*The challenge related to this activity was that the COVID vaccines were not available. However, I was still able to vaccinate elderly individuals with the shingles shot. Although, I wasn’t able to immunize the community with COVID vaccine I still felt like I was making the community healthier as a whole by vaccinating individuals against shingles. And I was also involved in checking patient in for their covid testing*”.

—Pharmacy Student A (class of 2023)

## 4. Discussion

The System was successful in the support of COVID-19 vaccination efforts in our community. This was possible due to many key factors. First, only minor modifications were needed to the existing system to incorporate COVID-19 immunization activities. Students, faculty, and preceptors did not have to learn a new process as they organized new vaccine activities. Second, strong collaborations were essential in creating and planning activities. For example, shortly after vaccine authorization by the FDA, there was a plethora of opportunities for SSPPS to support vaccine efforts through existing partnerships, such as experiential training agreements and our collaborative nature with UC San Diego Health (UCSDH), where many students complete experiential rotations and many faculty hold practice sites. In fact, UCSDH requested SSPPS support to help vaccinate the community at a large-scale clinic [7], and past collaborations along with personal connections were vital to ensuring SSPPS students and faculty were included in the scheduling and recruiting process, as well as troubleshooting issues. One such issue was ensuring UCSDH organizers were aware that SSPPS students were trained in vaccine administration, so they could be assigned to appropriate roles. In other examples of leveraging experiential training agreements, SSPPS students supported vaccinations at local independent pharmacies to help meet community demand and collaborated with large pharmacies that were immunizing high-risk populations, such as older adults in long-term care facilities. Overall, strong internal and external collaborations between SSPPS and partner organizations allowed for quick communication, resolution of issues, and the timely administration of COVID-19 vaccines.

Another key factor for success of the System was the engagement, motivation, and mobilization of SSPPS pharmacy students. Incoming SSPPS students are taught about professionalism and their role as members of the healthcare team. They also complete the American Pharmacists Association (APhA) Pharmacy-Based Immunization Delivery course at the start of their first year, enabling the entire student body to provide vaccines. These factors, along with an unprecedented need for immunizers in the local community, made students enthusiastic about supporting vaccine efforts. The level of involvement between class cohorts followed a predictable pattern. First-year students—trained to provide immunizations just 3 months previously—were involved in most of the vaccine effort. Other class cohorts, presumably with more responsibilities in school, work, and extracurricular events, made up a smaller portion of the overall student effort. Further, it was apparent that faculty did not have the bandwidth to organize every potential event. Allowing student organizations to take a lead role, with faculty oversight, provided an efficient system where students assumed most logistical responsibilities and was consistent with how other activities in the IPPE and/or CC courses were organized.

Based on review of self-reflections, we can conclude students encountered several challenges during COVID-19 activities that were associated with the Practical Issues domain of the BeSD Framework. These included logistical issues such as Availability, Ease of Access, and Service Quality. For example, efficient preparation of doses during vaccination clinics was noted, which has also been identified as a challenge at similar COVID-19 vaccination clinics [8]. SSPPS students provided evidence that they were able to overcome many of these challenges, such as utilizing their training to assist in preparing doses or providing additional vaccines to appropriate individuals. This provides some evidence that SSPPS students can apply their didactic training to identify immunization gaps and administer appropriate vaccines, even in settings that are focused on one vaccine (i.e., COVID-19). This also reinforces keeping immunization training towards the beginning of the curriculum, giving students ample opportunities to apply it. 

Our System was not without challenges. Rapidly changing information on vaccines and public health guidelines, plus modifications to pharmacy laws and regulations, made updating our student organizers and participants in a timely manner difficult. Several of these policies, such as increasing the ratio of pharmacists to interns when engaged solely in immunization-related activities, affected the logistics of our vaccine events. Student organizers rearranged student volunteers and preceptors to maximize immunizers based on these new rules. Additionally, at mass vaccination sites the requirement to notify a patient’s primary care provider after receipt of a COVID-19 vaccine was waived if certain conditions were met. These regulatory changes increased the burden for SSPPS faculty responsible for updating documents, forms, and other procedures pertinent to the System. Having a dedicated faculty member familiar with immunization practices and regulations—in our case, the Director of IPPEs—largely responsible for this aspect was beneficial in our System. However, the opportunity cost for dedicating a significant amount of time to ensuring all information was updated and accurate may have prevented the performance of higher functional duties of a clinical pharmacist such as patient care, mentorship, and education. 

Another challenge was understanding the extent to which students were participating with COVID-19 vaccines outside of events approved via the System. For example, many students provided vaccinations in their internship positions at community pharmacies or local health systems. Any activities completed during internships are not counted towards course credit, and therefore students would not submit these activities through the System. Further, students in the 2021 cohort provided vaccinations as part of their advanced pharmacy practice experiences (APPEs), which also was not captured by the System. Other students volunteered on their own time, with other organizations, and did not report the hours for class credit. Therefore, our total SSPPS efforts quantified here are likely under reported. Future iterations of the System should include ways to capture the full scope of efforts by all students.

The ability to be flexible and adaptive has been cited as a key factor for success of other COVID-19 vaccination efforts with pharmacy involvement [9]. We also discovered this was vitally important. Initially, some scheduled events were canceled due to lack of vaccine availability. This information often became available at the last minute, and communication to participating volunteers needed to be triaged quickly. To facilitate this, our System included recording the contact information for all student organizers and volunteers and designating responsible persons for communicating time sensitive information. Despite last minute changes or cancellations, students and preceptors continued to volunteer at future events.

The amount of data from the student’s reflection provides us with an opportunity to perform thematic content analysis, which will help inform key stakeholders on how to improve the vaccination experience. In this manuscript, we provided a preliminary summary of our qualitative investigation focusing on Practical Issues of the BeSD Framework [6]. Work is currently underway to formalize the qualitative assessment of these reflections using existing theoretical frameworks. This may result in improvements to the logistics of vaccination administration, education, and patient experience.

Other institutions may consider adopting a similar approach using their existing infrastructure to support vaccination efforts with partner organizations in their communities. By maintaining an approval mechanism for activities, leveraging existing partnerships, and fostering student engagement—this structure has the potential to serve many patients while providing quality learning experiences.

## 5. Conclusions

The System employed by SSPPS was efficient, flexible, and built on existing processes. Key factors for success included utilizing an existing activity approval system, leveraging strong collaborations with partner organizations, and robust engagement of student pharmacists. Further, student pharmacists overcame challenges encountered with vaccine activity logistics. Ultimately, the System contributed to the ability for SSPPS student pharmacists to maximize support for COVID-19 vaccination efforts.

## Figures and Tables

**Table 1:** Number of community vaccine effort (hours) by student class cohort.

Class Cohort	N	Total Hours
2021	2	23
2022	11	56
2023	31	315
2024	52	828
Total	96	1222

## Data Availability

Not applicable.
